# Sodium pertechnetate (Na99mTcO_4_) biodistribution in mice exposed to cigarette smoke

**DOI:** 10.1186/1471-2385-5-1

**Published:** 2005-04-11

**Authors:** Samuel S Valenca, Elaine AC Lima, Gláucio F Dire, Mário Bernardo-Filho, Luís Cristóvão Porto

**Affiliations:** 1 Departamento de Histologia e Embriologia, Universidade do Estado do Rio de Janeiro, Av. Prof. Manoel de Abreu 444 3° andar – Rio de Janeiro, RJ – 20551-170 Brasil; 2 Departamento de Biofísica e Biometria, Universidade do Estado do Rio de Janeiro, Av. Prof. Manoel de Abreu 444 3° andar – Rio de Janeiro, RJ – 20551-170 Brasil

## Abstract

**Background:**

The biological effects of cigarette smoke are not fully known. To improve our understanding of the action of various chemical agents, we investigated the biodistribution of sodium pertechnetate (Na^99m^TcO_4_) in mice exposed to cigarette smoke.

**Methods:**

Fifteen BALB/c male mice were exposed to the smoke of nine whole commercial cigarettes per day, 3 times/day, for up to 10 days to whole body exposure in a chamber. A control group of 5 BALB/c male mice was sham-smoked. One day later, the exposed and control groups of mice received (7.4 MBq/0.3 ml) of Na^99m^TcO_4 _before being killed at 30 min. Bones, brain, heart, intestine, kidney, liver, lungs, muscle, pancreas, spleen, stomach, testis and thyroid were weighed and these organs and blood radioactivity recorded with a gamma counter. The percentage per gram of tissue of injected dose (%ID/g) was determined for each organ.

**Results:**

Cigarette smoke significantly decreased (p < 0.05) the %ID/g in red blood cells, bone, kidney, lung, spleen, stomach, testis and thyroid of the exposed mice.

**Conclusion:**

The toxic effects of cigarette smoke reduced the Na^99m^TcO_4 _biodistribution.

## Background

Tobacco can be smoked in cigarettes, cigars, pipe, water pipes, or chewed. It is as an important cash crop, and has vast economical impact affecting the livelihoods of the farmers growing it, the companies that manufacture it, and the healthcare system that deals with the consequences of using it. Cigarette smoking (CS), the most popular method of smoking tobacco, is one of the most prevalent social habits practiced worldwide today [1]. The World Health Organization estimated that almost 1.1 billion people are smokers. Smoking has been identified as the leading preventable cause of death and disability in the world [2,3].

The development of diagnostic tools using radioisotopes is widely used in almost all hospitals nationwide. In nuclear medicine practice, physicians set norms for morphological or physiological function for each organ by diagnosing a large number of patients. For every procedure, there is diagnostic data for a range of normal variations familiar to physicians. Nuclear medicine practitioners interpret diseases on the basis of deviations from these limits [4]. Radioisotopes provide vital information to help diagnosis and therapy of various medical diseases. Data on tissue shape, function and localization within the body are relayed by use of one of various radionuclides, which can either be a free chemical species or covalently bound to part of a larger organic or inorganic moiety. These images are generated by the distribution of radioactive decay of the nuclide [5]. Technetium-99m (^99m^Tc) is the most frequently used radionuclide in diagnostic nuclear medicine procedures for a wide variety of diseases [6]. Various radiochemicals have been labelled with this nuclide. ^99m^Tc has a half-life of 6 h and emits γ-rays of 140 keV with an abundance of 90%. ^99m^Tc is primarily obtained from ^99^Mo/^99m^Tc generator and is eluted as sodium pertechnetate (Na^99m^TcO_4_). In this chemical form, it can be used to study brain and thyroid [7]. Intravenously administered ^99m^Tc-pertechnetate is loosely bound to plasma proteins and rapidly moves out of the intravenous compartment. The plasma half-time clearance is ~30 min. Approximately 30% of the administered dose is excreted within 24 h. The total urinary and faecal excretion of ^99m^Tc activity is about 50% in 3 days and up to 70% after 8 days. ^99m^Tc is also trapped by the thyroid gland and it passes into the small intestine. However in the brain, the blood-brain barrier prevents Na^99m^TcO_4_from entering brain cells [6,7].

The biodistribution and kinetics of radiochemicals can be altered by a variety of chemical agents, as is widely known [8,9]. Without knowing that these chemical agents are being used, the images can be affected to give poor organ visualization, possibly necessitating repeating the procedure, and resulting in unnecessary irradiation or even misdiagnosis [10]. The effect of CS on the biodistribution of radiochemicals has not been fully evaluated. The clearance of ^99m^Tc-pentetic acid aerosols is markedly increased in both sarcoidosis and other inflammatory lung diseases. However, the limitations of ^99m^Tc-pentetic acids include the fact that cigarette smoking will also cause increased clearance [11]. Moreover, the pulmonary uptake of ^99m^Tc-HMPAO (hexamethylprophylenoaminooxin) induced by smoking appears to be partially reversible after the cessation of smoking [12]. We evaluated the biodistribution as an experimental model to understand the action of various chemical agents [13-16]. This study deqals with possible changes in Na^99m^TcO_4 _biodistribution induced by the effects of CS in an animal model.

## Methods

Adult male BALB/c mice were housed, 5 per cage, in a controlled environment room, with light/dark cycle conditions (12 h light/12 h dark, lights on at 6 p.m.), for an acclimatization period of >3 weeks at an ambient temperature was kept at 25 ± 2°C. The animals had free access to water and food.

Male BALB/c mice (n = 15), weighting 20–22 g, were exposed to smoke-air mixture of commercial filtered Virginia cigarettes, 3 times/day for 1 (CS1d), 5 (CS5d) or 10 (CS10d) days by whole body exposure in an inhalation chamber. A control group (n = 5), sham-smoke (exposed to environment air), was also used. This protocol was performed twice with a total of 40 animals, which were weighed at the end of the experiment.

Each group of mice was placed in the inhalation chamber (40 cm long, 30 cm wide and 25 cm high), inside an exhaustion chapel. A cigarette was coupled to a plastic 60 ml syringe so that puffs could be drawn in and subsequently expelled into the exposure chamber. We aspirated one litre of smoke from one cigarette with this syringe (20 puffs of 50 ml) and immediately injected the puff into the chamber. The 5 animals of each group were maintained in this smoke-air condition for 6 min (~ 3 %), and the inhalation chamber was opened, by removing its cover, and the smoke evacuated for 1 min by exhaustion of the chapel. This cigarette exposition was repeated three times (3 × 6 min) with intervals of 1 min (exhaustion). We repeated this procedure 3 times per day (morning, lunch time and afternoon) resulting in a 54 min of CS exposition of 9 cigarettes. This protocol has been described by us elsewhere [17] and was an experimental protocol approved by the Instituto de Biologia Roberto Alcantara Gomes Animal Research Ethics Committee – UERJ.

Twenty-four hours after exposure, the mice received 0.3 ml of Na^99m^TcO_4 _(7.4 MBq) via the ocular plexus, and 30 min later, they were rapidly killed. Heart perfusion was performed with saline at constant pressure of 25 cm H_2_O to clear organs of blood for 5 min. The organs were isolated (bones, brain, heart, intestine, kidney, liver, lungs, muscle, pancreas, spleen, stomach, testis and thyroid), their weights determined, and the organs and blood sample radioactivity of Na^99m^TcO_4 _measured by a gamma-counter NaI (TI) (Cobra Auto-gamma, Packard Instrument Co.; Downers Grove, Illinois, USA). The percentage per gram of tissue of injected dose (%ID/g) was determined for each organ. Statistical analyses involved one-way ANOVA, followed by the Tukey-Kramer Multiple Comparisons Test, with the significance level being p < 0.05. InStat Graphpad software was used to perform statistical analysis (GraphPad InStat version 3.00 for Windows 95, GraphPad Software, San Diego Ca, USA).

## Results

The effects of CS on the biodistribution of Na^99m^TcO_4 _in isolated organs can first be seen in Table 1, which shows that CS decreased the %ID/g in red blood cells, bone, kidney, lung, spleen, stomach, testis and thyroid of the exposed mice. The greatest decrease was at CS10d in kidney (p < 0.001), spleen and testis (p < 0.01). In the stomach, CS exposure changes the %ID/g for CS1d, CS5d and CS10d (p < 0.05) were comparable with the controls. Table 2 shows that CS did not change the %ID/g of Na^99m^TcO_4 _significantly in brain, heart, intestine, liver, muscle and pancreas of exposed animals.

**Table 1:** Organs in which CS significantly changed (%ID/g) Na^99m^TcO_4 _biodistribution (mean ± SD).

**CS effects of Na^99m^TcO_4 _biodistribution in mice**				
**Organs**	**Groups**			
	
	**Control Mean ± SD**	**CS1d Mean ± SD**	**CS5d Mean ± SD**	**CS10d Mean ± SD**

**Red blood cells**	3.63 ± 0.24	2.73 ± 0.14*	2.33 ± 0.23**	2.74 ± 0.26*
**Bone**	0.26 ± 0.07	0.24 ± 0.05	0.15 ± 0.07	0.13 ± 0.05*
**Kidney**	0.89 ± 0.15	0.80 ± 0.10	0.62 ± 0.09**	0.51 ± 0.05***
**Lung**	1.72 ± 0.68	1.33 ± 0.28	1.28 ± 0.12	0.86 ± 0.04*
**Spleen**	0.36 ± 0.12	0.34 ± 0.06	0.22 ± 0.05	0.17 ± 0.05**
**Stomach**	5.66 ± 1.49	3.53 ± 0.59*	3.24 ± 1.24*	3.37 ± 0.77*
**Testis**	0.25 ± 0.06	0.19 ± 0.07	0.16 ± 0.03	0.12 ± 0.01**
**Thyroid**	1.07 ± 0.88	0.80 ± 0.47	0.47 ± 0.38	0.28 ± 0.23*

Statistical significance: (*) p < 0.05, (**) p < 0.01, (***) p < 0.001 when compared with control.

**Table 2:** Organs in which CS unchanged (%ID/g) Na^99m^TcO_4 _biodistribution (mean ± SD).

**CS effects of Na^99m^TcO_4 _biodistribution in mice**				
**Organs**	**Groups**			
	
	**Control Mean ± SD**	**CS1d Mean ± SD**	**CS5d Mean ± SD**	**CS10d Mean ± SD**

**Brain**	0.11 ± 0.02	0.14 ± 0.04	0.11 ± 0.01	0.10 ± 0.03
**Heart**	0.53 ± 0.24	0.61 ± 0.17	0.35 ± 0.12	0.48 ± 0.18
**Intestine**	0.91 ± 0.52	0.47 ± 0.21	0.72 ± 0.18	0.55 ± 0.39
**Liver**	2.32 ± 1.30	1.60 ± 0.21	2.07 ± 0.89	1.20 ± 0.37
**Muscle**	0.40 ± 0.35	0.14 ± 0.06	0.24 ± 0.21	0.09 ± 0.03
**Pancreas**	0.33 ± 0.28	0.16 ± 0.07	0.11 ± 0.03	0.13 ± 0.05

## Discussion

Cigarette smoke is a complex mixture of chemicals containing more than 4000 different constituents. Some of the compounds identified include pyridine alkaloids, such as nicotine, as well as ammonia, acrolein, phenols, acetaldehyde, N-nitrosamine; aromatic hydrocarbons (such as benzopyrene), combustion gases (e.g. carbon monoxide, nitrogen oxides, and hydrogen cyanide), trace metals, α-emitter radioactive elements such as polonium, radium, and thorium. Most of these compounds are produced by pyrolysis and distillation in the zone immediately behind the lighted tip of a cigarette, where the temperature reaches 950°C [18,19]. It has been estimated that undiluted mainstream smoke contains as much as 30 mg of tar and 5 mg of nicotine in an unfiltered regular tar cigarette or 0.5 mg tar and 0.05 mg nicotine in a filtered low-tar cigarette [20].

Radionuclides have been used to investigate diseases related to smoking [21,22]. In a recent study, Iwado et al. [23] demonstrated a decrease in the vasomotor response by PET (positron emission tomography) in the endothelium, in smokers. Moreover, acute CS can diminish cerebral blood flow, as was shown with technetium-^99m^-labelled ethylcysteine in single-photon emission tomography (SPET) on 10 healthy human volunteers [24]. However, there were no changes in biodistribution in the brain in our study. However, the blood-brain barrier prevents Na^99m^TcO_4 _from entering normal brain, and the radioactivity detected may represent a minimal amount of Na^99m^TcO_4 _from blood in brain circulation not cleared with our perfusion protocol.

Our experimental model showed that CS could affect %ID/g of Na^99m^TcO_4 _distribution in certain organs but not others. These changes vary according to the time of exposure; CS10d organs had lower %ID/g when compared to CS5d and CS1d, except for red blood cells, where there was lower %ID/g in CS5d. In this study, CS affected Na^99m^TcO4 %ID/g in red blood cells, bone, kidney, lung, spleen, stomach, testis and thyroid. It is noteworthy that some of these organs have also been associated with CS-induced cancer in humans [25].

Cigarette smoke can interfere with the ability of bone cells to participate in repairing and remodelling events [26]. This could be one of the mechanisms leading to the development of osteoporosis. Pathological analysis of the kidney by Cigremes et al. [27] has shown severe degeneration of this tissue with advanced hydrophobic degeneration of kidney tubules in the CS exposed animals.

Kidney radioactivity decreased with CS exposure as a function of time. There are two different effects of CS; first, less availability of Na^99m^TcO_4 _due to diminished blood flow to the kidney; and the second is increased permeability or secretion of Na^99m^TcO_4_. Further studies to determine the levels of Na^99m^TcO_4 _in the urine following CS exposure may conclude whether the decrease in radioactivity is as a result of one of these above changes.

Significant dependence of alveolar deposition on flow rate, but not lung function, was found in young non-symptomatic cigarette smokers and in older non-smokers with ^99m^Tc-polystyrene particles [28]. This could explain the significant decrease of Na^99m^TcO_4 _%ID/g in mouse lung at CS10d. Lungs are the main target of cigarette smoke [17], and a change in biodistribution of Na^99m^TcO_4 _in this experimental design was anticipated. Sopori et al. [29] have reported results on spleen cells from animals subjected to heavy doses of cigarette smoke which show a significant reduction in the natural killer cell-mediated proteolysis activity and a decreased response to concanavalin A.

There is a higher incidence of gastric cancer in smokers than non-smokers and cigarette smoking has also a strong association with colon cancer [30]. The uptake of Na^99m^TcO_4 _was significantly reduced in the stomach in our study, although not in the intestine. Technetium^99m ^is secreted via the stomach mucous and transferred into the intestine, which leads to an anticipated reduction of its radioactivity in intestine. Non-significant reduction of ^99m^Tc activity in the intestine is probably due to a great variability in radioactivity measured in this organ among animals.

Rajpurkar et al. [31] analysed chronic cigarette smoke on rat testis, and observed apoptosis, which may be one of the pathogenic mechanisms responsible for defective spermatogenesis in these animals, Fisher et al. [32] showed that chronic smokers have higher thyroxin levels and a lower thyroid-stimulating hormone level than non-smokers or former smokers. These results corroborate the effects on specific organs by CS, which validates this experimental design as a method to seek alterations of Na^99m^TcO_4 _biodistribution in mice.

The knowledge of radionuclide biodistribution due to chemical agents will help physicians avoid misinterpreting images and, thus from making incorrect diagnoses. Such information also helps to prevent or treat possible adverse reactions to radionuclides [15]. Therefore, developing models to study the interactions of chemical agents and radiopharmaceuticals will be of considerable help in understanding this problem. CS has many compounds and smoking habits vary in the population; a direct association between the CS effects and the biodistribution of ^99m^Tc has not previously been reported. However, we cannot comment on how CS changes the biodistribution in other radionuclides, radiochemicals and radiopharmaceuticals. The use of laboratory animals under controlled experimental conditions provides a good model to test the hypothesis that CS can change their biodistribution.

When comparing the sensitivity of CS in BALB/c mice to Na^99m^TcO_4 _biodistribution, we cannot exclude the possibility that C57BL6 mice will give better results in the future. When Na^99m^TcO_4 _is used as a powerful diagnostic tool in nuclear medicine for examining patients to assess the brain and thyroid, CS can cause alterations in the readings.

## Conclusion

CS induced changes of Na^99m^TcO_4 _biodistribution in exposed BALB/c mice. Na^99m^TcO_4 _distribution on kidney, lung, spleen, testis and thyroid decreased progressively with CS time exposure from 1 to 10 days. The Na^99m^TcO_4 _distribution in stomach and red blood cells was affected from the first day of CS exposure.

## Competing interests

The author(s) declare that they have no competing interests.

## Authors' contributions

**SSV **– carried out the experimental procedure with the mouse exposed to CS, performed the statistical analysis and write the manuscript.

**EACL **– carried out the experimental procedure with Na^99m^TcO_4 _(7.4 MBq) via the ocular plexus.

**GFD **– determined the radioactivity of the Na^99m^TcO_4 _in a counter NaI (TI)

**MB**-**F **– conceived of the study and participated in its design and coordination.

**LCP **– conceived of the study and participated in its design and coordination.

All authors read and approved the final manuscript.

## Pre-publication history

The pre-publication history for this paper can be accessed here:


